# Adapting primary care for new migrants: a formative assessment

**DOI:** 10.3399/bjgpopen17X100701

**Published:** 2017-01-09

**Authors:** Elizabeth Such, Elizabeth Walton, Brigitte Delaney, Janet Harris, Sarah Salway

**Affiliations:** 1 Research Fellow, School of Health and Related Research (ScHARR), University of Sheffield, Sheffield, UK; 2 GP and NIHR Clinical Lecturer, Academic Unit of Primary Medical Care, University of Sheffield, Sheffield, UK; 3 Research Associate, Academic Unit of Primary Medical Care, University of Sheffield, Sheffield, UK; 4 Senior Lecturer, ScHARR, University of Sheffield, Sheffield, UK; 5 Professor of Public Health, University of Sheffield, Sheffield, UK

**Keywords:** migrant, primary healthcare, general practice

## Abstract

**Background:**

Immigration rates have increased recently in the UK. Migrant patients may have particular needs that are inadequately met by existing primary care provision. In the absence of national guidance, local adaptations are emerging in response to these new demands.

**Aim:**

To formatively assess the primary care services offered to new migrants and the ways in which practitioners and practices are adapting to meet need.

**Design & setting:**

Online survey and case studies of current practice across primary care in the UK. Case studies were selected from mainstream and specialist general practice as well as primary care provision in the third sector.

**Method:**

Non-probability sample survey of primary care practitioners (*n* = 70) with descriptive statistical analysis. Qualitative case studies (*n* = 8) selected purposively; in-depth exploration of organisational and practitioner adaptations to services. Analysis is structured around the principles of equitable care.

**Results:**

Survey results indicated that practitioners focused on working with communities and external agencies and adapting processes of, for example, screening, vaccination, and health checks. Lack of funding was cited most frequently as a barrier to service development (*n* = 51; 73%). Case studies highlighted the prominence partnership working and of an organisational and practitioner focus on equitable care. Adaptations centred on addressing wider social determinants, trauma, and violence, and additional individual needs; and on delivering culturally-competent care.

**Conclusion:**

Despite significant resource constraints, some primary care services are adapting to the needs of new migrants. Many adapted approaches can be characterised as equity-oriented.

## How this fits in

High levels of immigration are placing additional demands on primary care services in England and elsewhere. A variety of local responses are emerging in England but these have not been described or evaluated to-date. Using survey data from 70 primary care practitioners and eight case studies this study provides important early insights into local adaptations. The adaptations documented aim to address wider social determinants, trauma, and violence, and additional individual needs; and also to deliver culturally-competent care. Resource constraints are identified as a common obstacle to delivering satisfactory care to new migrants.

## Introduction

Migration reached its highest recorded levels in 2015 with a net 334 000 people arriving and staying in the UK.^1^ This presents a series of challenges to primary care, not least because new migrants form a diverse population, some of whom face particularly vulnerable circumstances.^2–4^ More generally, new arrivals may be disadvantaged because of unfamiliarity with health systems and processes, language, and cultural differences, and discriminatory behaviours and processes.^5–7^

Primary care can adapt at various levels; from healthcare practitioner knowledge and skills, through to organisational cultures, to wider interorganisational linkages and policy.^5,7–10^ Some provision is recognised internationally as good practice: high quality interpreting services; comprehensive health care with service integration; intersectoral collaboration; training and mentorship for healthcare professionals; and enhanced organisational cultural competence.^11–13^ Providing high quality and equitable primary health care is, however, challenging in a resource-squeezed climate.^14,15^ The situation is compounded in the UK by a policy environment dominated by concerns about migrant legal status and entitlements rather than guidance on what counts as good practice.^1–18^ Indeed, although there are some notable exceptions,^19,20^ little explicit national guidance is provided on how existing services should be adapted to meet need. Evidence suggests differing interpretations of entitlement guidelines at the front line^21^ and a range of emergent local responses to migrant healthcare needs.

This study provides early insights into how primary care practices are responding with the dual aims of identifying the key issues being faced and useful strategies that may warrant formal piloting and evaluation.

## Method

The study was conducted in two phases in winter 2015–2016: an online survey and in-depth case studies. The survey was developed to collect data on where and how primary care was being adapted to need and to identify potential case studies. A targeted distribution approach was adopted across the sector. Alongside questions about perceptions of patient population change and adaptations to services, responders were asked to provide contact details for telephone follow-up. From the follow-ups, eight case studies were selected to represent a range of specialist and mainstream primary care providers.^22^ ‘Specialist GP’ practices offered bespoke services to marginalised populations such as refugees, asylum seekers and undocumented or irregular migrants. They had a defined remit to serve these groups. ‘Mainstream’ practices offered a generalised service and would adapt it to meet need, often without any additional funding.

Survey analysis, telephone discussions, and existing research informed the following case study themes: how and why services were adapted; developments over time; and obstacles and enablers of innovation. One group interview (with three GPs from the same city) and seven in-depth interviews (four GPs, one specialist nurse, one clinic manager, and one programme lead) were conducted. Analyses of organisational and practitioner documents (*n* = 11; staff training materials, organisational reports, and practitioner protocols) complemented the interviews.

Descriptive statistics were used for the survey analysis. Case study data were first subjected to inductive thematic coding.^23^ The study then drew on Browne and colleagues’^14^ framework to assess the equity-oriented nature of the adaptations being offered. The analytical approach provided insight into practices as well as underlying drivers for adaptation.

## Results

### Survey

Seventy full, individual survey responses were achieved. GPs (family physicians) were the largest group of responders (*n* = 40; 57%) and the data are skewed towards the North of England with the North West and Yorkshire and the Humber accounting for 44 (63%) responses: 84% identified that their migrant patients had increased in numbers in the past 5 years, with 35 (50%) reporting a rapid increase. Responses highlighted diversity in the migrant populations served. Sixty-five (93%) reported treating migrant patients from two or more global regions in the past 5 years. Sixty (86%) reported patients who were refugees and asylum seekers.

Fifteen (21%) responders reported no service adaptations in response to new migrants among their patient population. Of these, five reported a steady increase in migrants in their practice over the past 5 years and eight reported a rapid increase.

Interpreter services were offered according to 64 (91%) responders: 47 (67%) offered longer appointment times and 20 (29%) had patient involvement groups for new arrivals in their area. Table 1 highlights how provision was adapted in terms of staffing, partnership working and the service offer. Over half (*n* = 38; 54%) reported working with other organisations or agencies (such as charities, community groups, or police) to help provide services for new migrants.

**Table 1.** Adaptations reported to meet the needs of new migrant populations

Adaptation		*n*	%	
Staffing	Volunteer community/peer health workers	24	34	
	Cultural competency training	26	37	
	Community health nurses/other health professionals	21	30	
Partnership working	Signposting to support organisations, such as welfare support advice	51	73	
	Strategic coordination with other agencies, such as housing associations, local schools	27	39	
Service offer	Outreach activity long- or short term	25	36	
	Community-embedded services	24	34	
	Altered/atypical opening hours/appointment times	22	31	
	Co-located services, such as with social care	19	27	
	Clinical specialist services beyond usual remit, such as mental health	24	34	
None of the above		15	21	

Percentages subject to rounding; more than one response was possible.

Many barriers to service development were reported (Table 2) and 64 practitioners (91%) reported one or more such barriers.

**Table 2.** Reported barriers to meeting needs of new migrant populations

	Barrier to developing services	*n*	%
Resources and funding	Lack of funding	51	73
	Insecurity of funding	33	47
Population factors	Patients needs are too varied to account for them all	17	24
	Not knowing patients needs	13	19
	Populations change too frequently to meet need	9	13
Capacity of staff	Lack of time	45	64
	Personal fatigue/burnout/capacity	24	34
	Lack of staff	30	43
	Lack of skills in the team to address needs	12	17
Rules and regulations	Lack of clarity about NHS charging rules	15	21
	Commissioning rules	14	20
	Lack of clarity about migrant patient eligibility	10	14
No barrier identified		6	9

Percentages subject to rounding; more than one response was possible.

### Case studies

The eight case studies included five mainstream GP practices and three specialist practices (serving refugees, asylum seekers, trafficked people, and undocumented migrants), across Scotland,^2^ Northern England,^5^ and London.^1^ Adaptations were varied and related to staffing, what and how services were delivered, partnerships, and patient-provider interactions. Adaptations could broadly be categorised as aiming to address social determinants of health, needs associated with trauma and violence, other special healthcare needs, and culturally-competent care (Box 1).

Practices varied in the extent of adaptations reported, but in all cases, there was evidence of a commitment at both an individual and an organisational level to improving the lives of marginalised people:

**Box 1.** Examples of primary healthcare practice mapped to the dimensions of equity-oriented service (adapted from Browne and colleagues 2012)

	Addressing wider social determinants	Addressing trauma and violence	Addressing additional, specific individual needs	Delivering culturally-competent care
**Staffing**	Dedicated health professionals such as health visitors for some migrant groups (S)	Training materials on trauma, violence and insecurity (S) Secondary trauma counselling for health professionals (S) Attention to non-threatening physical environment (S) Specialist nurses for patients with traumatic histories (S) GPs with specialisms in asylum seeker/refugee health (S)	Specialist health practitioners experienced in working with marginalised/migrant patients (S)	Volunteer community health advocates Face-to-face interpreters at drop-in clinics Support staff with community languages Face-to-face interpreters wherever possible (S) Cultural competency training for staff (S)
**Service offer**	Routine interdisciplinary case reviews (S)	Protocols for responding to issues related to trauma and violence such as FGM Family clinics for vulnerable women and children (S)	Drop-in clinics for specific populations Local vitamin D and hepatitis B protocols Outreach services for those not attending clinics (S) Follow-up consultations with health professional after first contact (S) Tailored protocols for assessment of new arrivals (S)	
**Partnership working**	Social prescribing Signposting such as to welfare support Close links with secondary care such as infectious disease Co-location with specialist organisations such as mental health; asylum/refugee support (S) Routine multi-agency working such as housing and schools (S)	Working with local non-statutory services (such as Rape Crisis) to refer patients for support	Engagement in tailored projects such as Roma Health Projects Pre-arrival preparation systems for people arriving under managed migration schemes (S)	Development of patient involvement groups with new migrant representation (S)
**Patient–provider interaction**	GPs advocating for migrant patients such as supporting immigration applications Holistic assessment of patients needs and resources (S)	Empowering practice to support traumatised patients (such as peer support) (S) Mental health integrated into patient assessments (S) Detailed medical histories (S)	Longer appointment times to allow for interpreter use and assessment of complex cases (20 mins or up to 30 minutes [S])	Adapted written prescription guidelines to aide medication adherence Translated health education materials

(S) indicates adaptations offered only by the specialist services.

‘I think in terms of values, everyone sees the work that we do in serving vulnerable groups as a privilege. Although there’s lots of challenges, it doesn’t feel like it’s a problem —  I think seeing things in terms of opportunities … partly that’s been about recruiting the right people. Growing the team. Supporting each other and taking hold of new opportunities.’ (‘Specialist service’ GP)

‘The practice manager and the partners are positive towards this group of people. They are keen to service this population well, and learn more, training for their staff, that sort of thing, that is absolutely crucial … If everyone has a positive attitude that’s really helpful, especially those in charge.’ (‘Mainstream’ GP)

Organisational commitment was made explicit in some policy statements and also demonstrated through active involvement in local strategy groups and communities of practice, advocacy for system change, and practice sharing with central government by some specialist services. Notable were the number and type partnerships across the case studies including charities, community groups, police, employment and welfare advice agencies, wellbeing projects, local authorities, secondary care services, housing associations, other GP practices, schools, and refugee agencies. This underlying commitment, and the range of adaptations on offer, suggest that many of these services could be characterised as ‘equity oriented’ as defined by Browne and colleagues.^8^ (Box 1).

Service adaptations were constrained, particularly by the funding environment which was perceived as insufficient and insecure. This was particularly the case for ‘mainstream’ services when standard service models could not accommodate specific, additional needs:

‘The one thing that we really struggle with and feel don’t have the capacity is for, is the huge number of patients from, not only the asylum seekers but also the Chinese and various other backgrounds that aren’t aware of their immunisation status. Public health advice being that if you’re not sure then you should give them an entire primary immunisation course, and we just don’t have the resources or capacity to do that. 1) To establish what their immunisation status is, and 2) to actually do a whole course again. That’s probably a big issue from a public health point of view, not just our, our point of view.’ (Mainstream GP)

In mainstream services, enhancing services beyond existing resource envelopes and outside prevailing target systems (such as the Quality and Outcomes Framework) was a topic for debate at practice level and outcomes were variable. Some enhancements were simply ‘absorbed’ or resources reorganised (for example, using healthcare assistants to do health assessments instead of practice nurses); others were balanced against other resource considerations and sometimes additional funding applications were made:

‘[*GP and the senior partner*] were just talking about, say for example, at Asylum Health, they do a very big starter consultation where they document a lot of life events, like, for example, FGM, torture, or other things that might have happened, PTSD, and I was talking about whether we could do that in our new patient assessment and the response was "well, if it’s gonna take time from other patients, then …" So, there’s a resource thing.’ (Mainstream GP)

‘The migrant health screening I did manage to push through, so we do the Hep bloods and HIV and stuff, but other than that I’ve not managed to change anything for the funding.’ (Mainstream GP)

Additional and sometimes challenging workloads also raised issues of ‘burn-out’ and stress for health professionals, leading one of the case studies to introduce ‘life coaching’ for staff and another to adopt secondary trauma team debriefing similar to those techniques used in conflict areas.

## Discussion

### Summary

The survey highlighted the diverse nature of the migrant population being served. Despite commonly reported resource constraints, around 80% of responders reported adaptations to service approach and provision for new migrants. The survey and case studies demonstrated that practitioner and organisational commitment to equity drove adaptations, which centred on addressing wider social determinants, trauma, and violence and additional needs, and providing culturally-competent care. Examples of adaptation demonstrated creative ways of meeting need, particularly partnership working. Nevertheless, insufficient funding and staff stress were serious concerns.

### Strengths and limitations

This was a formative exploratory study covering a small selected sample of the 9000 GP practices in the UK.^24^ As such, the findings cannot be generalised.

Nevertheless, in the context of poor practice-level data on migration^25^ limited evidence on the healthcare needs of new migrants, and few documented examples of primary care practice in this area, the study findings do provide some early insights.

### Comparison with the existing literature

The findings suggest that approaches to serving new migrant populations in primary care are in many ways consistent with those proposed in previous research, particularly in the area of cross-agency collaboration and community-fed service adaptation.^11,12,26^ They also highlight that resourcing adapted services remains a challenge, particularly in the context of existing high clinical workload in general practice.^27^

### Implications for research and practice

Although not unproblematic, some very common adaptations to practices (such as signposting or longer appointment times) may be compatible with current resource structures of non-specialist GP provision. Others present a significant resource and resilience challenge time, funds, and personal ability to cope. It is necessary to better understand these challenges, how they are experienced and what the outcomes are for practitioners and patients in the contemporary context of primary care. Some barriers are generic in that they reflect systemic changes to general practice (clinical, managerial, or workforce)^28^ while others are specific to serving migrant patients in terms of practitioner skills and understandings of, for example, patient eligibility. Some of the practices of specialised services warrant further exploration to establish transferability to mainstream general practice. What is evident is that primary care is able to make innovations despite experiencing barriers although limits are clearly evident in the negotiation of over-stretched resources.
